# Comparative validation of a microcapsule-based immunoassay for the detection of proteins and nucleic acids

**DOI:** 10.1371/journal.pone.0201009

**Published:** 2018-07-20

**Authors:** Sujit Kumar Verma, Anja Karin Albrecht, Verena Siebecke, Gerd Klöck, Tatiana A. Kolesnikova, Sebastian Springer

**Affiliations:** 1 Department of Life Sciences and Chemistry, Jacobs University, Bremen, Germany; 2 City University of Applied Sciences, Bremen, Germany; Michigan State University, UNITED STATES

## Abstract

To detect and study diseases, research and clinical laboratories must quantify specific biomarkers in the plasma and urine of patients with precision, sensitivity, and cost-effectiveness. Newly developed techniques, such as particle-based immunoassays, must be validated in these terms against standard methods such as enzyme-linked immunosorbent assays (ELISAs). Here, we compare the performance of assays that use hollow polyelectrolyte microcapsules with assays based on solid plastic beads, and with standard microplate immunoassays. The polyelectrolyte microcapsules detect the disease biomarker beta-2 microglobulin with a fifty-fold increase in sensitivity than polystyrene (PS) beads. For sequence-specific nucleic acid detection, the oligonucleotide-coated microcapsules exhibit a two-fold lower increase in sensitivity over PS beads. The microcapsules also detect the presence of a monoclonal antibody in hybridoma supernatant at a fifty-six-fold increase in sensitivity compared to a microplate assay. Overall, polyelectrolyte microcapsule-based assays are more sensitive for the detection of protein and nucleic acid analytes than PS beads and microplate assays, and they are viable alternatives as a platform for the rapid quantitative detection of analytes at very low concentrations.

## 1 Introduction

For diagnosis and monitoring of disease, the accurate, sensitive and early measurement of biomarkers is essential. The rapid and sensitive detection of protein analytes, a critical technique in diagnostic science, can be achieved with the help of specific antibodies. Although enzyme-linked immunosorbent assays (ELISAs) are currently the most popular method of antibody-based assays, there are more sensitive, robust, and economical alternatives. For example, while ELISAs can detect one analyte [1], particle-based immunoassays allow simultaneous detection of multiple analytes in a single well [2–7]. In such particle-based assays, the use of flow cytometry has increased the sensitivity and largely removed the need for washing steps [8]. While most particle-based assays currently use solid beads made of polystyrene (PS) or other plastics, we have recently introduced the use of hollow polyelectrolyte microcapsules [9]. Their porous surface can be modified with large amounts of antibodies [10–12], and their great physico-chemical stability aids the development of assays that are fast and robust in a broad range of experimental conditions. One can also attach nucleic acids to the microcapsule surface for the sequence-specific detection of nucleic acids [9].

Polyelectrolyte microcapsules are produced through assembly of alternating layers of positively charged (such as polyallylamine hydrochloride, PAH) and negatively charged (such as polyacrylic acid, PAA) long-chain molecules onto micrometer-sized calcium carbonate (CaCO_3_) cores, driven by electrostatic attraction [13,14], and subsequent covalent cross-linking (for example with 1-ethyl-3-(3-dimethylaminopropyl) carbodiimide, EDC) [15]. The resulting shells are resistant to even extreme pH and salt conditions [16–19]. To attach antibodies and other proteins, their lysine side chains are activated with EDC and N-hydroxysulfosuccinimide (sulfo-NHS) and then coupled to the carboxylate groups of the outermost PAA layer [20]. The CaCO_3_ cores are left in place for all assay manipulations, which makes it easier to centrifuge and wash the particles and are then removed with ethylenediaminetetraacetic acid (EDTA) just before reading the assay so that the resulting hollow microcapsules remain in suspension (microcapsules have approximately the same density as the buffer) for analysis in the flow cytometer. This allows samples to be analyzed for a longer period of time, especially in diagnostics laboratories where many samples are continuously processed.

This study describes the validation and application of a highly sensitive and selective microcapsule-based immunoassay for the detection of proteins and nucleic acids [9]. We compare polyelectrolyte microcapsules with commercially available polystyrene (PS) beads in the detection of a protein (the disease marker human beta-2 microglobulin, hβ_2_m) and a nucleic acid. We find that the microcapsule-based assay is ultra-sensitive and detects hβ_2_m in the femtomolar range, whereas the detection limit of the PS bead-based assay for the same protein is in the nanomolar range. The microcapsule assay might thus be uniquely useful to detect hβ_2_m at an early stage of diseases such as renal failure. We also demonstrate the use of protein A-coated microcapsules to monitor the production of a monoclonal antibody by a hybridoma. Our results show that polyelectrolyte microcapsule-based immunoassays are robust techniques for protein and nucleic acid detection and can serve the requirements for a sensitive, robust and optical read-out immunoassay [21].

## 2 Materials and methods

### 2.1 Materials

5–6 μm diameter calcium carbonate (CaCO_3_) particles (Cat No. PL-CA6-10g) were purchased from PlasmaChem (Berlin, Germany). Poly(allylamine hydrochloride) (PAH) sodium salt (Cat. No. 283223), N-Hydroxysulfosuccinimide sodium salt (sulfo-NHS) (Cat. No. 56485), ethylenediaminetetraacetic acid (EDTA) disodium salt dehydrate (Cat. No. A3553) were purchased from Sigma-Aldrich. Poly(acrylic acid) (PAA) sodium salt (Cat. No. 18611) was purchased from Polysciences (Hirschberg, Germany). 1-(3-Dimethylaminopropyl)-3-ethylcarbodiimide hydrochloride (EDC) (Cat. No. A10807) was purchased from Alfa Aesar (Heysham, UK). Protein A (Cat. No. 21181) and FITC-labeled streptavidin (Cat. No. 21224) were purchased from Thermo Scientific. Sodium chloride (Cat. No. A4857), disodium hydrogen phosphate (Cat. No. A3905), sodium carbonate (Cat. No. A1881), sodium bicarbonate (Cat. No. A1940), potassium chloride (Cat. No. A2939), potassium dihydrogen phosphate (Cat. No. A3095) streptavidin (Cat. No. A1495), bovine serum albumin (BSA) (Cat. No. A1391), sodium azide (Cat. No. A1430), and 2-morpholinoethanesulfonic acid (MES) (Cat. No. A1074), were purchased from AppliChem (Darmstadt, Germany). Polyclonal goat anti-mouse antibody (Cat. No. A11001) and goat anti-rabbit antibody (Cat. No. A11008) labeled with Alexa Fluor 488 were purchased from Invitrogen, and polyclonal rabbit anti-human β_2_m (Batch 5511) was purchased from Nordic Immunology. RPMI media (Cat. No. 880175) was purchased from Lonza. Tween 20 (Cat. No. 9127) was purchased from Roth. 0.45 μm syringe filters (Cat. No. 16555K) and spin filters of 0.8 μm pore size (Cat. No. VK01P042) were purchased from Sartorius Stedim Biotech. High-binding black 96 well flat polystyrene microplates with clear bottom (Cat. No. 655097) were purchased from Greiner Bio-One (Frickenhausen, Germany). Carboxylated silica (SiO_2_) beads of 1.01 μm and 2.12 μm (Cat. No. AR756 and AR833), poly(methylmethacrylate) (PMMA) beads of 1.02 μm and 2.08 μm (Cat. No. AR830 and AR145) and polystyrene (PS) beads of 1.20 μm and 2.35 μm (Cat. No. A1482 and B874) sizes were purchased from microparticles (Berlin, Germany). Murine monoclonal hybridomas BBM.1 (against hβ_2_m) [22] and W6/32 (against human MHC class I) were from Alain Townsend (Oxford University) and Peter Cresswell (Yale University); the antibodies were purified with standard methods using protein A agarose beads. Human beta-2 microglobulin (hβ_2_m) was produced in *E*.*coli*, folded *in vitro* as described [23], and then purified by size exclusion chromatography on a Superdex 200 10/30 GL column (GE Biosciences). The oligonucleotides were supplied by Eurofins Genomics (Munich, Germany; **S1 Table**).

### 2.2 Preparation and crosslinking of core-shell particles

50 mg of CaCO_3_ particles were suspended in 2 mL of Milli-Q water, sonicated for 5 min, and then washed three times by centrifugation (all washes were done at 3000 rpm for 2 min with Milli-Q water). After the washes, 2 mL of PAH (2 mg mL^-1^ in 0.5 M NaCl, pH 7.0) were added and incubated for 10 min, continuously shaking at 1200 rpm. The particles were then washed three times, and 2 mL of PAA (2 mg mL^-1^ in 0.5 M NaCl, pH 7.0) were added and incubated for 10 min to adsorb the second layer of polyelectrolyte. In total, two layers of PAH and PAA each in alternating layers were adsorbed. The (PAH/PAA)_2_ polymer layers were crosslinked with 10 mg mL^-1^ EDC in MES buffer (0.1 M MES in 0.5 M NaCl, pH 6.0) overnight while shaking at 1200 rpm. The resulting core-shell particles were washed three times with ice-cold Milli-Q water.

### 2.3 Functionalization of (PAH/PAA)_2_ polymers and beads with proteins

The carboxyl groups on the (PAH/PAA)_2_ polymers and the beads were surface-activated by incubating them with 500 μL of freshly prepared 0.4 M EDC/0.1 M sulfo-NHS mixture in phosphate buffered saline (PBS, pH 7.2), shaking at 1200 rpm at room temperature (RT) for 1 h. After surface activation, particles were washed three times with PBS (pH 7.2). The surface-activated particles were then modified with adaptor proteins by incubating them either with 50 μg of protein A or streptavidin in 500 μL PBS (FITC-streptavidin was titrated to get the optimal concentration needed to coat the surface of the beads), pH 7.2 at RT overnight, shaking at 1200 rpm. All particles were washed three times with Milli-Q water to remove any unbound protein. Residual NHS esters were quenched by incubating the particles in 500 μL of 50 mM Tris-Cl (pH 8.8) at RT for 30 min. Before measurement in the flow cytometer, the core-shell particles were resuspended in 0.2 M EDTA (pH 7.2) to dissolve the CaCO_3_ core, thoroughly washed three times with Milli-Q water, and collected using spin filters at 800 rpm for 15 seconds.

### 2.4 Proof of binding: Antibodies

Three samples of the microcapsules and the different PS beads were taken, of which one sample was used as a background control. The other two samples were surface-activated with EDC/sulfo-NHS as described in section 2.3, washed, and then incubated with 50 μg protein A in 500 μL PBS, pH 7.2 each at RT overnight. Out of them, one sample was incubated with 10 μg of BBM.1 antibody in 1 mL PBS (pH 8.2) for 2 h, followed by the secondary GαM-AF488 (AF488- labeled polyclonal goat anti-mouse) antibody (0.2 μg in 1 mL PBS, pH 7.2) for 30 min. Last sample was incubated with GαM-AF488 (0.2 μg in 1 mL PBS, pH 7.2) for 30 min (control). All samples were washed three times with Milli-Q water after every step of incubation. All particles were then measured in the flow cytometer for AF488 fluorescence.

### 2.5 Proof of binding: Oligonucleotides

Microcapsules and PS beads were surface-activated as described in section 2.3. One sample was taken for setting the background signal. The other samples were incubated with 50 μg streptavidin in 500 μL 1x PBS (pH 7.2) overnight shaking at 1200 rpm at RT. The streptavidin-coated PS beads were then incubated with different concentrations (1 nM– 1000 nM) of biotinylated and Cy5-labeled oligonucleotide Oligo1 in 1 mL PBS (pH 7.2) for 2 h. All samples were washed with Milli-Q and then measured in the flow cytometer for Cy5 fluorescence.

### 2.6 Detection of hβ_2_m in PBS

Core-shell particles were prepared and cross-linked as described in section 2.2. The core-shell particles and the PS beads were then surface-modified with protein A as described in section 2.3. The protein A-coated particles were incubated with 10 μg BBM.1 antibody (murine monoclonal anti-hβ_2_m) in 1 mL PBS (pH 8.2) for 2 h, followed by incubation with the analyte hβ_2_m at different concentrations (10^−3^–10^5^ pg mL^-1^ for core-shell particles and 10^−3^–10^6^ pg mL^-1^ for PS beads) in PBS (pH 7.2) for 1 h [24]. The hβ_2_m-bound particles were washed thoroughly to remove all unbound hβ_2_m and blocked with 1% BSA for 45 minutes in 1x PBS (pH 7.2). The samples were washed three times to remove non-specifically bound protein, followed by incubation with 0.3 μg polyclonal rabbit anti-hβ_2_m (Rαhβ_2_m) in 1 mL PBS (pH 7.2) for 2 h. Finally, the particles were incubated with 0.2 μg detector antibody GαR-AF488 (AF488- labeled polyclonal goat anti-rabbit antibody) in 1 mL PBS (pH 7.2) for 30 min. As a negative control, BBM.1-modified particles were incubated with Rαhβ_2_m and GαR-AF488 antibodies but without analyte. Particles were washed three times with Milli-Q water after every step of incubation. The core-shell particles were dissolved with 0.2 M EDTA (pH 7.2). All particles were then measured in the flow cytometer. As a background control, crosslinked particles were used.

### 2.7 Detection of nucleic acids in PBS

Core-shell particles were prepared and crosslinked as described in section 2.2. The core-shell particles and the PS beads were then surface-modified with streptavidin as described in section 2.5. The streptavidin-coated particles were incubated with 50 nM biotinylated Oligo2 in 1 mL PBS (pH 7.2) for 2 h shaking at 1200 rpm at RT. For the analyte dose response, the Oligo2-modified particles were incubated with different concentrations of the analyte Oligo3 (10^−1^–10^3^ nM) in 1 mL PBS (pH 7.2) for 1 h, shaking at 1200 rpm. All the particles were then washed three times with Milli-Q water to wash off unbound analyte. The particles were incubated with 200 nM FITC-Oligo5 (detector) in 1 mL PBS (pH 7.2) for 30 min. As negative controls, 200 nM FITC-Oligo5 was added directly to the crosslinked or streptavidin-coated particles or to the Oligo2-modified particles in the absence of the analyte Oligo4 (**S1 Table**). The core-shell particles were dissolved with 0.2M EDTA (pH 7.2). All particles were then measured in the flow cytometer. As a background control, crosslinked particles were used.

### 2.8 Flow cytometry and plate spectroscopy

Flow cytometry data were acquired on a CyFlow Space flow cytometer (Partec) using green (488 nm) and red (638 nm) lasers and analyzed using FlowJo (FlowJo Enterprise). All plates were read on an Infinite M1000 plate reader (TECAN) with the excitation wavelength at 488 nm and the emission wavelength at 519 nm.

### 2.9 Detection of BBM.1 antibody

Core-shell particles were prepared, crosslinked, and surface-modified with protein A as described in section 2.2 and 2.3 [9]. High-binding 96-well black well plates were coated with 100 μg mL^-1^ protein A in carbonate buffer (pH 9.6) overnight at 4°C and then blocked with 1% (w/v) BSA overnight. The protein A-coated core-shell particles were incubated in 1 mL with purified murine monoclonal BBM.1 either at a concentration of 2×10^−3^–3×10^1^ μg mL^-1^ in 1 x PBS (pH 7.2) or at a concentration of 1×10−3–3×10^1^ μg mL^-1^ in complete RPMI media for 2 h shaking at 1200 rpm. The 96-well plates were also incubated in 100 μL with purified murine monoclonal BBM.1 at a concentration of 1.9×10^−2^–1×10^1^ μg mL^-1^ either in 1x PBS (pH 7.2) or at a concentration of 5×10^−4^ – 1x10^1^ μg mL^-1^ in complete RPMI media for 2 h shaking at 400 rpm. All samples were then incubated with the detector antibody GαM-AF488 (0.2 μg in 1 mL PBS, pH 7.2) for 30 min shaking at RT. All samples were triply washed either with Milli-Q (for core-shell particles) or with PBST (PBS with 0.01% Tween 20, for 96-well plates) after every step of incubation. Samples incubated with detector antibody GαM-AF488 alone serve as negative controls. The cores of the core-shell particles were dissolved with 0.2 M EDTA (pH 7.2), and the microcapsules were then measured in the flow cytometer. The 96-well plates were read in the TECAN Infinite reader.

### 2.10 Sample collection during BBM.1 hybridoma culture

BBM1 hybridoma was grown in RPMI media (10% fetal calf serum with glutamine), and eight different fractions of the hybridoma were collected at eight different days (day 2, 5, 9, 12, 16, 19, 23, and 26). All samples were centrifuged at 2000 g for 10 minutes, and the collected supernatants were filtered through a 0.45 μm syringe filter. The samples were then stored at 4°C with 0.01% sodium azide.

### 2.11 Monitoring the growth of the BBM.1 antibody in hybridoma supernatant

Microcapsules and 96-well plates were coated with protein A as described in section 2.3. Murine monoclonal BBM.1 hybridoma cells were grown in a T-225 flask. The media was not replaced, and samples were collected at different days of culture and the cells counted (**S2 Table**). The collected fractions were centrifuged, filtered through a 0.45 μm syringe filter, and stored at 4°C with 0.01% NaN_3_. 1 μL and 2 μL of the different hybridoma fractions collected during different days of culture were added to both the core-shell particles (in a total volume of 2 mL) or to the 96-well plates (in a total volume of 100 μL/well) and incubated for 2 h at RT. All samples were then washed and incubated with detector antibody GαM-AF488 (0.2 μg in 1 mL PBS, pH 7.2) for 30 min shaking at RT. All samples were washed three times either with Milli-Q (for core-shell particles) or with PBST (PBS with 0.01% Tween 20, for 96-well plates) after every step of incubation. Samples with detector antibody GαM-AF488 alone served as negative controls. The core-shell particles were dissolved with 0.2 M EDTA (pH 7.2) and then measured.

### 2.12 Data analysis

Dose-response curves were fitted using a four-parameter logistic curve obtained with a nonlinear regression fitting procedure in the GraphPad Prism 7.0 analytical software (GraphPad, La Jolla, CA). The limit of blank (LoB), limit of detection (LoD), and limit of quantification (LoQ) values for each analyte were calculated using the following equations: LoB = mean blank + 1.645 x (SD of blank); LoD = LoB + 3 x (SD of a low-concentrated sample); LoQ = LoB + 10 x (SD of an accurately quantified sample). The calculated values were interpolated from the corresponding calibration curves using GraphPad Prism.

## 3 Results and discussion

### 3.1 Preparation and characterization of microcapsules and beads

Commercially available beads made of polystyrene (PS), silica (SiO_2_), or poly(methylmethacrylate) (PMMA) are currently used for detection of analytes and for multiplexing studies that are read out by flow cytometry [1,25]. We wanted to directly compare these beads with polyelectrolyte microcapsules with respect to their binding capacity for antibodies and their sensitivity for protein and nucleic acid analytes.

**Fig 1:**
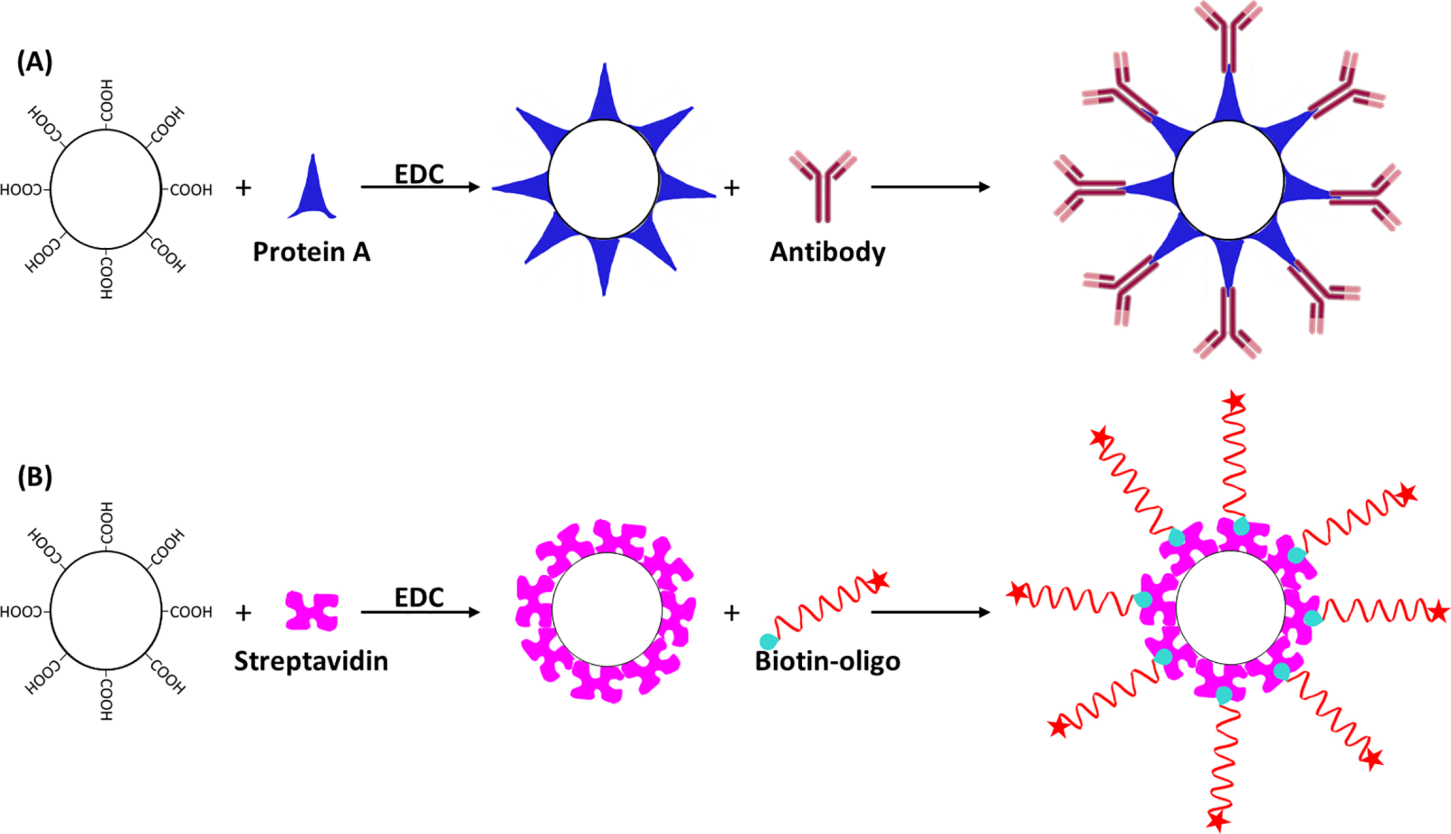
**Coating of microcapsules and beads with proteins or nucleic acids:** The carboxylic acid groups in the outmost layer of the microcapsules were surface-activated by EDC/sulfo-NHS. Then, either protein A was attached onto the activated surface for oriented binding of antibodies (A), or streptavidin was immobilized for the binding of biotinylated oligonucleotides (B). The coating of commercially available beads was performed in the same way. Drawings are not to scale.

First, we tested which of the commercially available beads are most suitable for binding antibodies. Solid beads used for flow cytometry-based assays must be in the size range between 1 and 2.5 μm to remain in suspension (whereas hollow microcapsules, whose density is equal to that of water, are not subject to this limitation). Beads made of SiO_2_ (1.0 and 2.1 μm), PMMA (1.0 and 2.1 μm), and PS (1.2 and 2.4 μm), which had been modified to carry carboxylic acid groups on their surface, were surface-activated with EDC/sulfo-NHS and then coated with *Staphylococcus aureus* protein A. The coated beads were then incubated with the murine monoclonal antibody BBM.1 (**Fig 1A**). To quantify antibody binding to the beads, they were treated with a fluorescence-labeled secondary antibody and then measured by flow cytometry. Among all the beads tested, the 2.35 μm PS beads bound the largest amount of the BBM.1 antibody per bead (**S1 Fig**). We therefore used these beads for comparison with the polyelectrolyte microcapsules.

### 3.2 Microcapsules are better tools for the detection of protein analytes

**Fig 2:**
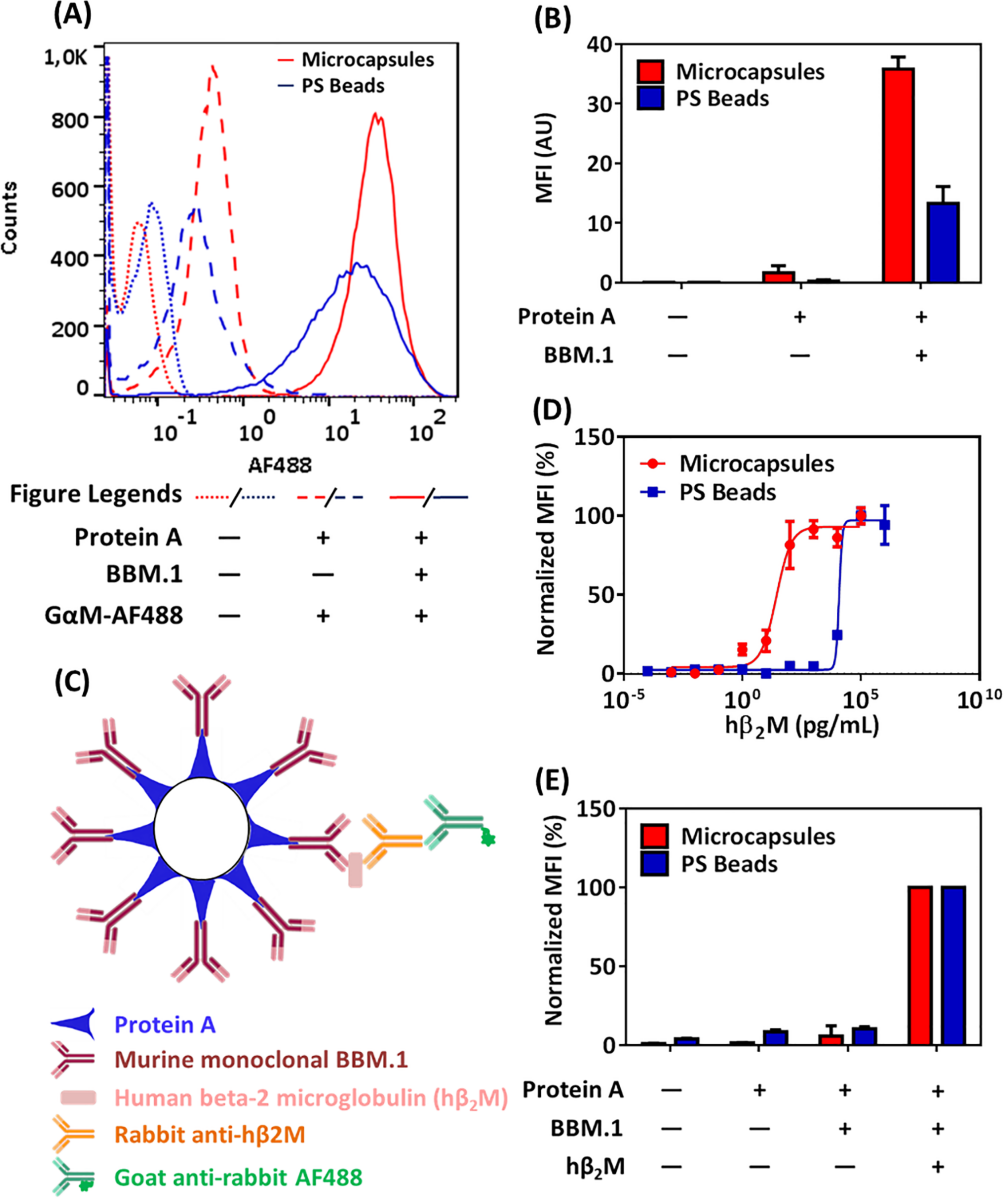
**Comparison of 6 μm microcapsules and 2.35 μm PS beads for binding of antibody and for the detection of a protein analyte:** (A+B) Detection of the capture antibody, BBM.1, on microcapsules and beads with fluorescently labeled goat anti-mouse antibody (GαM-AF488) by flow cytometry. (A) shows a representative experiment, (B) the average of two experiments with standard deviations (SD). (C) Schematics for the detection of human beta-2 microglobulin (hβ_2_m). The capture antibody, BBM.1, is immobilized on the protein A-coated microcapsules/PS beads. BBM.1 antibody binds specifically to hβ_2_m, which is sandwiched by the polyclonal rabbit anti-hβ_2_M (Rαhβ_2_M) antibody. The sandwich is then detected by adding AF488 labeled goat anti-rabbit (GαR-AF488) antibody. (D) Detection of hβ_2_m in PBS. Dose-response curves for the assay performed as in (C) with microcapsules or PS beads. MFI values are normalized to the maximum values. Error bars are SD (n = 3). Invisible error bars are smaller than the size of the marker. (E) Control samples of hβ_2_m plotted as histograms. Experiments were performed as in (C). Samples with analyte (10^5^ pg mL^-1^ for microcapsules and 10^6^ pg mL^-1^ for PS beads) were used as positive control and for normalization, which was done individually for microcapsules and PS beads. Error bars are SD (n = 3).

Next, we compared the binding of antibodies to microcapsules and PS beads. We produced 6 μm microcapsules and confirmed the correct assembly of the polymers with zeta potential measurements (**S2 Fig**). In the BBM.1 antibody-binding assay, the microcapsules showed an average mean fluorescence intensity (MFI) of 33.54 versus 12.23 for the PS beads (*p*-value = <0.00001), demonstrating that the microcapsules bound 2.7 times more antibody (**Fig 2A and 2B**). We then tested both microcapsules and PS beads in a sandwich immunoassay for the protein analyte, human beta-2 microglobulin (hβ_2_m), which is measured in the plasma to detect cancer and in the urine to detect renal failure [26]. In this assay, the BBM.1 antibody on microcapsules or PS beads binds hβ_2_m, which is then detected with polyclonal rabbit anti-hβ_2_m antibody, followed by fluorescently labeled anti-rabbit secondary antibody (**Fig 2C**) [9]. A dose-response study comparing the microcapsules and the PS beads showed clearly that the microcapsules detected hβ_2_m with much higher sensitivity than the PS beads (**Fig 2D**). For the microcapsule-based assay, a distinct fluorescence signal was detected already at the very low analyte concentration of 1 pg mL^-1^, compared to 100 ng mL^-1^ for the PS beads. The limit of detection (LoD, i.e., the lowest analyte concentration that can be feasibly detected) was 99 fg mL^-1^ for the microcapsules, whereas that of the PS beads was 5.0 ng mL^-1^ (**Table 1**); thus, the microcapsules exhibited higher sensitivity, which may be crucial for hβ_2_m detection in the urine. The microcapsule-based assay also had a better signal-to-noise ratio (**Fig 2E**).

**Table 1:** Analytical performance of hβ_2_m and oligonucleotide detection using microcapsule and PS beads: Best fit values were obtained with a four-parameter fit equation. Limit of blank (LoB), limit of detection (LoD) and limit of quantification (LoQ) were determined as described in the materials and methods.

	hβ_2_m in PBS		Oligo3 in PBS	
	Microcapsules	PS beads	Microcapsules	PS beads
**Bottom**	4.01	2.38	4.14	-0.24
**Top**	92.9	97.1	92.9	94.8
**Hill Slope**	1.38	6.44	6.23	1.75
**R**^**2**^	0.97	0.99	0.98	0.97
**LoB**	32 fg mL^-1^	3.8 ng mL^-1^	7.5 X 10^−9^ M	4.8 X 10^−9^ M
**LoD**	99 fg mL^-1^	5.0 ng mL^-1^	1 X 10^−8^ M	5.9 X 10^−9^ M
**LoQ**	2.1 pg mL^-1^	8.7 ng mL^-1^	12.4 X 10^−9^ M	16.5 X 10^−9^ M
**Concentration Tested**	10^−3^ to 10^5^ pg mL^-1^	10^−4^ to 10^6^ pg mL^-1^	10^−10^ to 10^−6^ M	10^−10^ to 10^−6^ M

### 3.3 Microcapsules exhibit similar sensitivity to PS beads for the detection of nucleic acids

**Fig 3:**
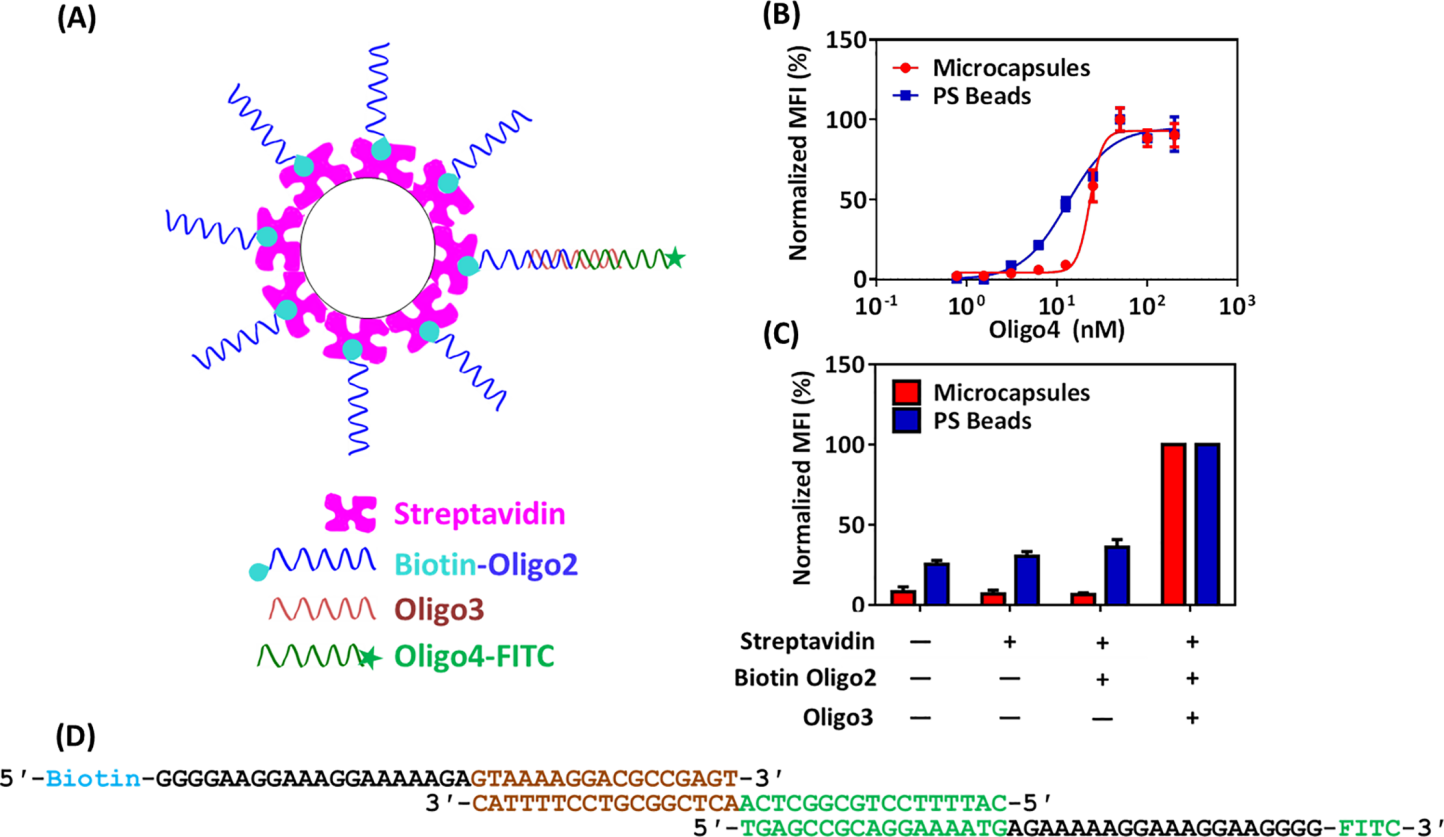
**Comparison of microcapsules and PS beads for the detection of nucleic acids:** (A) Schematics. The anchor, Biotin-Oligo2, is attached to streptavidin-coated microcapsules or PS beads andhybridizes specifically to its complementary sequence on the analyte and on Oligo3, which in turn hybridizes to its complementary sequence on the detector, Oligo4-FITC. (B) Analyte dose-response of the assay for streptavidin-coated microcapsules and PS beads measured by flow cytometry. MFI values were normalized by the maximum value. The error bars are SD (n = 3). Invisible error bars are smaller than the size of the marker. (C) Background controls for the assay in (B). MFI values were normalized to the maximum value. The error bars indicate the SD (n = 3). (D) Hybridization scheme of the three oligonucleotides used in the current assay. Biotin-Oligo2 hybridizes to the 17 complementary nucleotide bases (brown) of analyte Oligo3, and the remaining free 17 bases of the analyte hybridize to the detector Oligo4-FITC (green).

Rapid and sequence-specific detection of nucleic acids is another proposed application of particle-based assays. Thus, in the next set of experiments, we compared microcapsules and PS beads for the detection of short nucleic acid sequences [9]. Biotinylated anchor oligonucleotides are attached to streptavidin-coated microcapsules (or PS beads). The nucleic acid analyte, for example a single-stranded plasmid, an RNA transcript, or an oligonucleotide, binds to the anchor oligonucleotide through specific base pairing. A detection oligonucleotide, which is complementary to a free sequence in the analyte and labeled with a fluorescent dye, is then bound and detected by flow cytometry (**Fig 3A**).

We first determined the optimal concentrations of streptavidin and anchor oligonucleotide required to functionalize the surface of PS beads (**S3 Fig**). The covalently immobilized streptavidin on the PS beads was functional since it bound fluorescently labelled biotinylated oligonucleotides with a three- to five fold increase in the signal over the background (**S3 Fig**). For microcapsules, we have previously reported a tenfold increase under similar conditions[9].

We then compared the sensitivity of microcapsules and PS beads for the detection of an oligonucleotide analyte **(Fig 3D**) in a dose-response study (**Fig 3B and 3C**). The polyelectrolyte microcapsules and the PS beads showed similar sensitivity with LoD values of 1.0 x 10^−8^ M and 5.9 x 10^−9^ M, respectively. Importantly, the microcapsule-based assay had a lower background signal in the absence of the analyte than the PS beads (**Fig 3C**).

We conclude that polyelectrolyte microcapsules are suitable for highly sensitive protein and nucleic acid assays, and that for protein assays, they outperform the commercially available solid beads. **Table 1** contains the complete validation data for both the protein and the nucleic acid assays [27,28].

### 3.4 Monitoring antibody production during hybridoma culture

We next tested the microcapsule assay in a practical application, monitoring antibody production in the supernatant of antibody-producing hybridoma cells, and we compared its performance with that of a standard 96-well plate sandwich immunoassay that followed the same detection principle. The microcapsules (or plates) are coated with protein A, which then binds the analyte antibody, which is then sandwiched with fluorescently labeled anti-mouse antibody and measured by flow cytometry (or in a plate reader).

**Fig 4:**
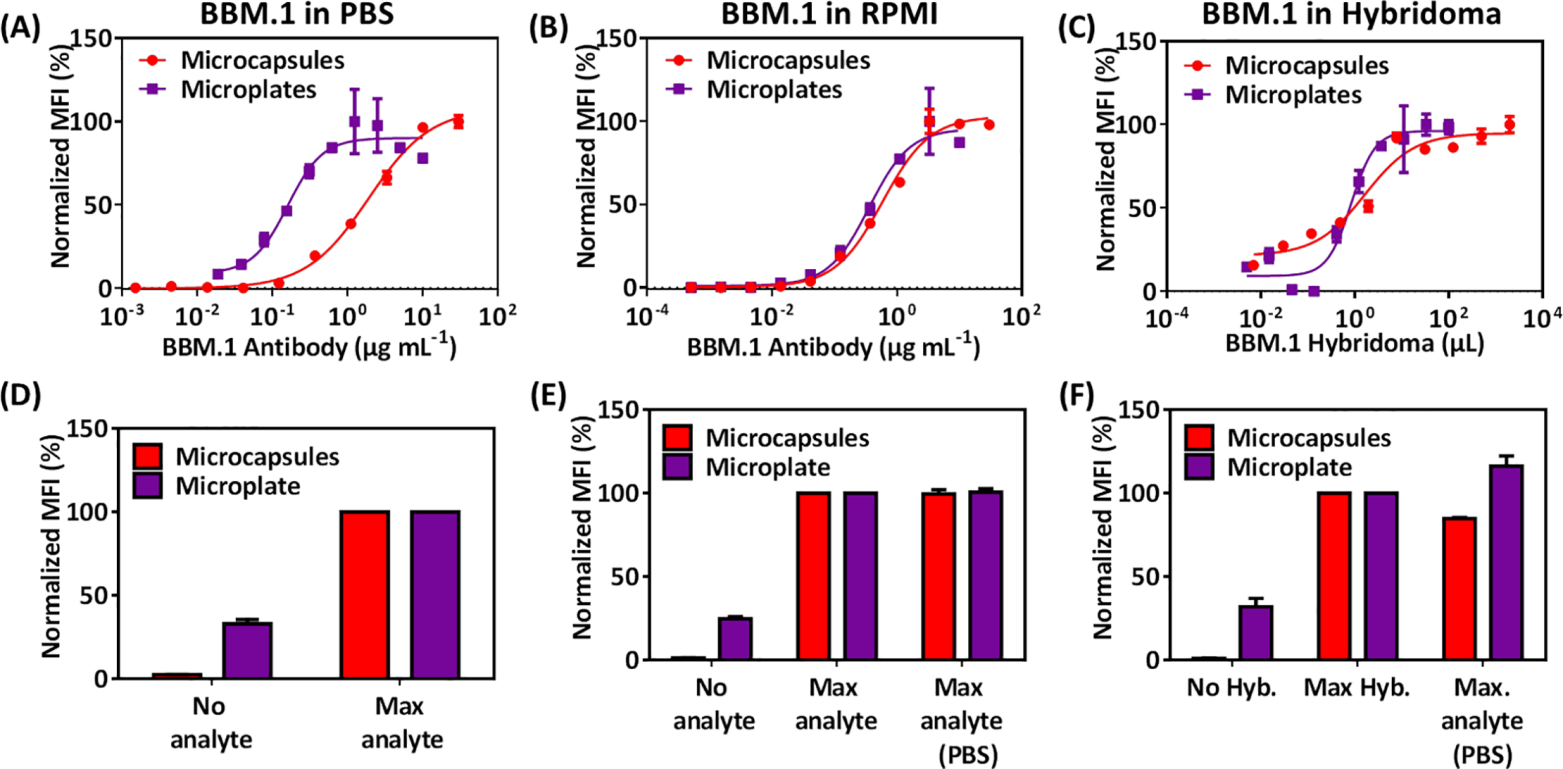
**Comparison of microcapsule and microplate assays in the detection of the BBM.1 antibody in hybridoma supernatant:** (A-C) Analyte dose-response curves for the BBM.1 antibody in PBS (A), RPMI (B), and dilutions of hybridoma supernatant (C). Microcapsules or plates were coated with protein A. After binding of the analyte, samples were incubated with detector antibody GαM-AF488 and measured either by flow cytometry (microcapsules) or in a plate reader (microplates). LoDs are: in (A) 26 ng mL^-1^ for microcapsules and 24 ng mL^-1^ for plates; in (B) 7 ng mL^-1^ for microcapsules and 54 ng mL^-1^ for plates; in (C) 2.8 nL mL^-1^ for microcapsules and 158 nL mL^-1^ for plates. See Table 2 for complete data. (D-F) Background controls and comparisons for the curves above. For (D) and (E), 'Max analyte' is the largest amount of analyte used in the assay above (20 μg mL^-1^ for microcapsules and 10 μg mL^-1^ for the microplate). For (F), 'Max Hyb.' is the largest volume of hybridoma used in the assay above (2000 μL for microcapsules and 100 μL for microplate). For (E) and (F), 'Max. analyte (PBS)' is 30 μg μL^-1^ in PBS. MFI values were normalized by the highest value (A-C), the 'Max analyte' value (D-E), and the 'Max Hyb.' value (F). Error bars are the SD (n = 3). Invisible error bars are smaller than the size of the marker.

For both assays, we first recorded dose-response curves with purified antibody in PBS. The plate-based assay seemed more sensitive (**Fig 4A**), but the LoDs were identical (26 ng mL^-1^ and 24 ng mL^-1^ for the microcapsules and the microplates, respectively; **Table 2**) because of the much lower background signal of the microcapsule-based assay (**Fig 4D**).

**Table 2:** Analytical performance of BBM.1 detection using microcapsule and microplate for BBM.1 hybridoma: Best fit values were obtained with a four-parameter fit equation. LoB, LoD, and LoQ were determined as described in the materials and methods.

	BBM.1 in PBS		BBM.1 in RPMI		BBM.1 in Hybridoma	
	Microcapsules	Microplates	Microcapsules	Microplates	Microcapsules	Microplates
**Bottom**	0.41	8.67	0.13	0.91	21.4	9.28
**Top**	108	90.02	103	95.6	94.9	96.3
**Hill Slope**	1.05	1.88	1.15	1.33	0.83	1.71
**R**^**2**^	0.99	0.92	0.99	0.97	0.95	0.95
**LoB (mL**^**-1**^**)**	21 ng	13 ng	5 ng	21 ng	0.9 nL	124 nL
**LoD (mL**^**-1**^**)**	26 ng	24 ng	7 ng	54 ng	2.8 nL	158 nL
**LoQ (mL**^**-1**^**)**	69 ng	125 ng	38 ng	118 ng	13.6 nL	882 nL
**Concentration Tested**	2x10^-3^ to 3x10^1^ μg mL^-1^	1.9x10^-2^ to 1x10^1^ μg mL^-1^	1x10^-3^ to 3x10^1^ pg mL^-1^	5x10^-4^ to 1x10^1^ pg mL^-1^	7x10^-3^ to 2x10^3^ μL	1x10^-2^ to 1x10^2^ μL

In RPMI medium with 10% fetal calf serum (FCS), which replicates the composition of the hybridoma supernatant medium, the dose-response curve of both assays looked similar (**Fig 4B**), with the LoD again significantly lower for the microcapsule assay (7 ng mL^-1^ and 54 ng mL^-1^ for the microplate assay; **Table 2**) due to the much lower background of the microcapsule assay (**Fig 4E**).

To measure the antibody concentration in the hybridoma supernatant, we titrated the supernatant either onto the protein A-coated microcapsules or into the protein A-coated 96-well plates, followed by washing and incubation with fluorescently labeled secondary antibody (**Fig 4C**). Both assays measured the same antibody concentration, approximately 330 μg mL^-1^, in the hybridoma supernatant sample, with the microcapsule assay showing a much better signal-to-noise ratio (**Fig 4F**). Interestingly and more remarkably, the LoD value for the hybridoma supernatant titration was again much lower for the microcapsules (2.8 nL mL^-1^ vs. 158 nL mL^-1^ for the plates).

The results of the hybridoma experiment demonstrate that the microcapsule-based assay is more sensitive than the 96-well plate assay under conditions where all other reagents, including antibodies and buffers, were identical. The better LoD values for the microcapsule assay can be attributed to the lower background signals and better values between the replicates of the sample for normalized data (**S3 Table)** and for non-normalized data **(S4 Table)**, since the calculation of LoD also considers the standard deviation between the samples [27]. The validation data for both assays are in **Table 2**.

Finally, we used our microcapsule-based assay to monitor the production of BBM.1 antibody during the course of a hybridoma culture, again comparing with the plate assay. Supernatant samples were collected at different days of culture and measured by either assay (**S4 Fig**). Both assays monitored equally well the production of antibody, which reached a plateau on day 5.

## 4 Conclusion

We show here for the first time the validation of a microcapsule-based immunoassay for the detection of protein and nucleic acid analytes compared to bead-based and microplate-based assays. Over the past decade, multiple bead- or particle-based fluorescent assays have been developed. They are especially attractive since they can be multiplexed, *i*.*e*., they allow detection of multiple analytes from limited amounts of sample. We have shown here that polyelectrolyte microcapsules[9] can be used to build sensitive and powerful particle-based assays for the detection of proteins and nucleic acids; especially, in the detection of protein analytes, microcapsules are more sensitive than a similar assay based on PS beads (**Fig 2D**).

The microcapsule-based assays presented in this study have several advantages over the plate or bead assays that are currently in use. First, with respect to the immobilization of the first layer of protein (such as protein A for antibodies or streptavidin for biotinylated oligonucleotides), microcapsules and beads have the common advantage over plates that proteins can be covalently attached through EDC/sulfo-NHS chemistry, which prevents the leaching of protein into the assay solution. This may, in fact, account for the lower background signal, the better signal-to-noise ratio, and therefore the lower LoD observed with microcapsules as compared to microplates (**Fig 4**).

Second, for the covalent attachment of proteins, microcapsules are more efficient than commercially available solid beads: first, they bind at least twice as much functional protein A than PS beads (**Fig 2A**), probably because the high porosity of the microcapsule wall offers a larger surface area. Second, our microcapsules have the advantage over filled beads that they contain CaCO_3_ cores that can be dissolved. The cores make them heavy, *i*.*e*., easy to handle at every step of the assay, since they can be sedimented in a microcentrifuge (3000 rpm for two minutes). But after the cores are dissolved at the end of the assay, the hollow microcapsules, which now have approximately the same density as the buffer, can be read conveniently and reproducibly in the flow cytometer. In contrast, commercially available PS, SiO_2_, or PMMA beads settle rapidly at the bottom of the tubes, which makes flow cytometry difficult and error-prone. This is especially important if the assay is performed in a high-throughput format, where individual samples may wait some time to be read. The synthesis of the CaCO_3_ microparticles that we use as cores can be performed in any analytical laboratory from sodium carbonate and calcium nitrate [29,30]; they are also commercially available. The use of EDTA to dissolve the cores might cause difficulties with antibodies that depend on divalent ions to bind their antigens; it might be necessary to optimize the dissolution protocol by choosing a different chelator and/or adding back the necessary ions.

Third, like other particle-based assays, the microcapsule-based assay can be used for multiplexing with different antibodies attached to different sets of capsules. Such capsule sets can have different sizes or fluorescent colors, which are either attached to the capsule walls or contained inside (*e*.*g*., coupled to dextran), and the sets can easily be distinguished in the flow cytometer by gating on size or fluorescence.

Fourth, and perhaps most importantly, our microcapsule assay is simple and robust and shows a higher sensitivity for proteins, with an LoD in the fg mL^-1^ range, while commercially available bead-based assays detect protein biomarkers in the pg ml^-1^ concentrations [2–5,31,32], and traditional ELISA assays for proteins have LoD values in the ng ml^-1^ range [33].

The increased sensitivity of the microcapsules in the detection of hβ_2_m that is demonstrated here has a potentially important practical application. In the blood, hβ_2_m serves as a prognostic marker for several cancer types such as multiple myeloma and lymphoma [34–36], with its concentration increased when disease occurs. The concentrations of hβ_2_m in the blood (1.4–2.5 μg mL^-1^) [37] can still be measured with conventional assays, but the currently available rapid bioassays cannot detect hβ_2_m in the urine, where an increase over the very low normal concentration of 30 ng mL^-1^ is a marker for renal failure [26]. Thus, a microcapsule-based assay might be crucial for the rapid early detection of renal failure, which accompanies a variety of diseases [38]. We believe that for other protein markers of disease, similar microcapsule-based assays can be constructed.

For the sequence-specific detection of nucleic acids, microcapsules show similar sensitivity to the commercially available PS beads and very high specificity for individual sequences[9]. This suggests that microcapsule-based assays can be used to detect single-stranded nucleic acids such as microRNAs, which are blood markers of cancer in 'liquid biopsies' [39], or antibiotic resistance genes from denatured samples for the rapid identification of multidrug-resistant bacteria. For such applications, it is important to develop test kits that do not require a PCR reaction that consumes time and complicates the analysis. This is an exciting direction for future research.

## Supporting information

S1 Fig Determination of antibody immobilization efficiency of commercially available carboxylated beads. (DOCX)

S2 Fig Monitoring of the production of the polyelectrolyte microcapsules by zeta potential measurement after each adsorption step. (DOCX)

S3 Fig Determination of streptavidin and oligonucleotide optimal concentrations. (DOCX)

S4 Fig Monitoring the production of BBM.1 antibody in hybridoma supernatant. (DOCX)

S1 Table Overview of oligonucleotides including abbreviation, sequence, length of the sequence and its function. (DOCX)

S2 Table Cell counts for BBM.1 hybridoma during culture. Aliquots of cell culture supernatant were collected and counted on the respective days.
(DOCX)

S3 Table Normalized values of the hybridoma experiments performed with microcapsules and microplates with their respective concentrations, average and standard deviation. (DOCX)

S4 Table Non-normalized values of the hybridoma experiments performed with microcapsules and microplates with their respective concentrations, average and standard deviation. (CSV)
