# Supplementary material for: Comparative validation of a microcapsule-based immunoassay for the detection of proteins and nucleic acids
Source: PLoS One. 2018 Jul 20;13(7):e0201009. doi: 10.1371/journal.pone.0201009 (PMC6054379; doi:10.1371/journal.pone.0201009)
Supplement: S1 Fig — (DOCX) [file pone.0201009.s001.docx]

**
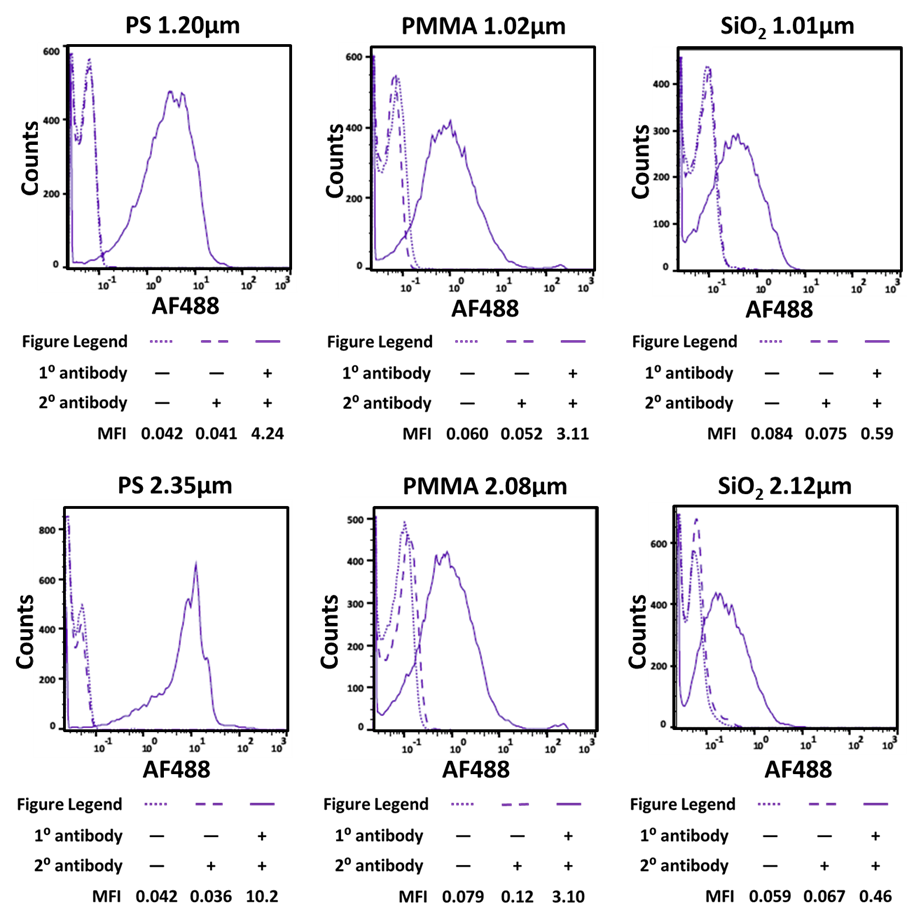
**

**S1 Fig. Determination of antibody immobilization efficiency of commercially available carboxylated beads:** Murine monoclonal BBM.1 antibody (1° antibody) was added to protein A-coated PS, PMMA, and SiO_2_ beads. The BBM.1 antibody was then detected by goat anti-mouse secondary antibody coupled to Alexa Fluor 488. Fluorescence of the beads was measured by flow cytometry.
