# Supplementary material for: Comparative validation of a microcapsule-based immunoassay for the detection of proteins and nucleic acids
Source: PLoS One. 2018 Jul 20;13(7):e0201009. doi: 10.1371/journal.pone.0201009 (PMC6054379; doi:10.1371/journal.pone.0201009)
Supplement: S2 Fig — (DOCX) [file pone.0201009.s002.docx]

#
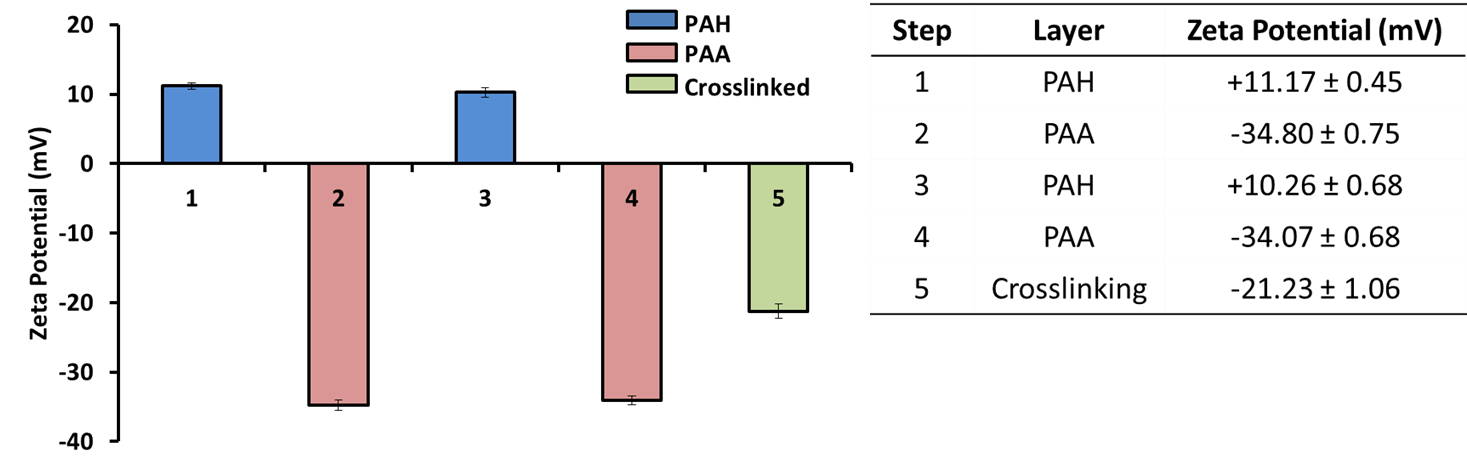


**S2 Fig. Monitoring of the production of the polyelectrolyte microcapsules by zeta potential measurement after each adsorption step**: Microcapsules were layered onto 6 µm CaCO_3_ particles. Error bars indicate the SD (n=3).
