# Supplementary material for: Comparative validation of a microcapsule-based immunoassay for the detection of proteins and nucleic acids
Source: PLoS One. 2018 Jul 20;13(7):e0201009. doi: 10.1371/journal.pone.0201009 (PMC6054379; doi:10.1371/journal.pone.0201009)
Supplement: S3 Fig — (DOCX) [file pone.0201009.s003.docx]

**
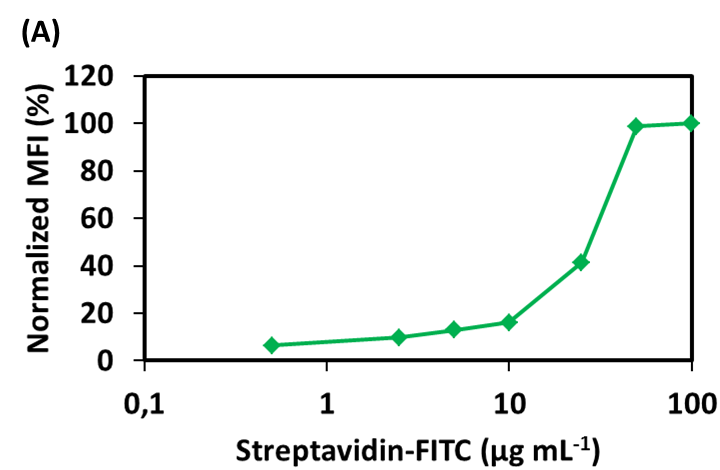

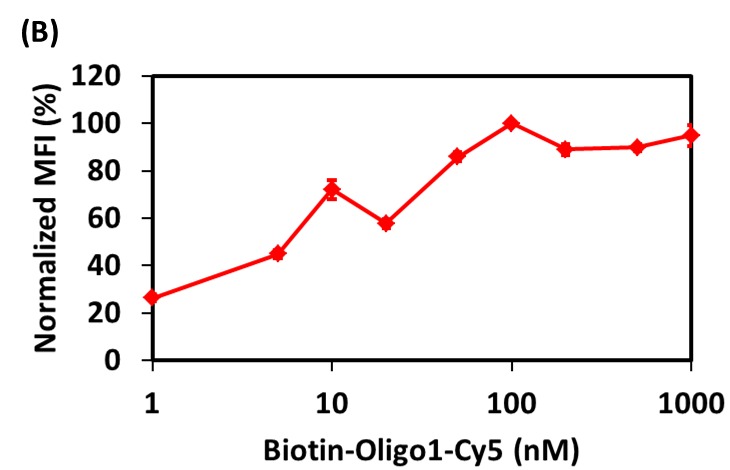
**

**S3 Fig. Determination of streptavidin and oligonucleotide optimal concentrations:**

1. **Titration of streptavidin.** Different concentrations of FITC-streptavidin were immobi­lized on the PS bead surface via EDC/sulfo-NHS chemistry. MFI values were normalized by the maximum value.
2. **Titration of anchor oligonucleotide:** Biotinylated Cy5-labeled oligo1 was immobilized to the streptavidin functionalized PS beads at different concentrations. MFI values are normalized by maximum value; error bars indicate the (SD; n=3).
