# Supplementary material for: Comparative validation of a microcapsule-based immunoassay for the detection of proteins and nucleic acids
Source: PLoS One. 2018 Jul 20;13(7):e0201009. doi: 10.1371/journal.pone.0201009 (PMC6054379; doi:10.1371/journal.pone.0201009)
Supplement: S4 Fig — (DOCX) [file pone.0201009.s004.docx]

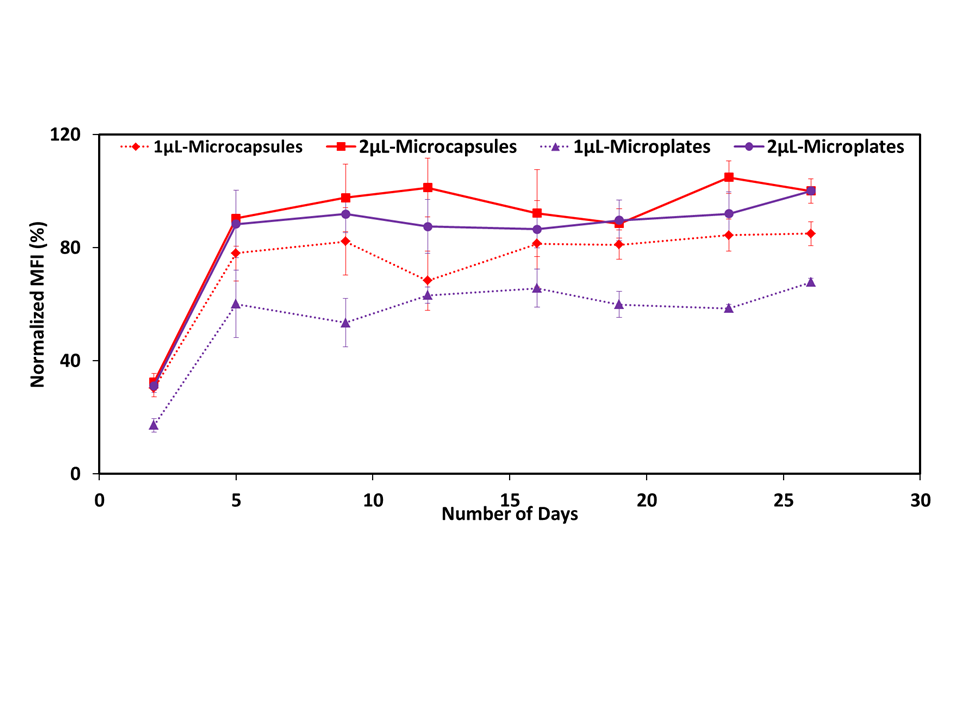


**S4 Fig. Monitoring the production of BBM.1 antibody in hybridoma supernatant:** Protein A-coated microcapsules and microplates were incubated with 1 µL and 2 µL hybridoma supernatants that were collected from different days of culture. Binding of the BBM.1 antibody was measured through binding of the detector antibody GαM-AF488. MFI values were normalized for each assay by the final value of the 2 µl curve. The error bars indicate the SD (n=3). Invisible error bars are smaller than the size of the marker.
