# Supplementary material for: Comparative validation of a microcapsule-based immunoassay for the detection of proteins and nucleic acids
Source: PLoS One. 2018 Jul 20;13(7):e0201009. doi: 10.1371/journal.pone.0201009 (PMC6054379; doi:10.1371/journal.pone.0201009)
Supplement: S1 Table — (DOCX) [file pone.0201009.s005.docx]

**S1 Table.** Overview of oligonucleotides including abbreviation, sequence, length of the sequence and its function.

**
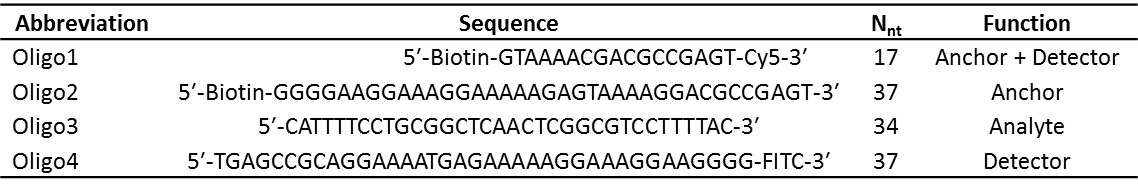
**
