# Supplementary material for: Comparative validation of a microcapsule-based immunoassay for the detection of proteins and nucleic acids
Source: PLoS One. 2018 Jul 20;13(7):e0201009. doi: 10.1371/journal.pone.0201009 (PMC6054379; doi:10.1371/journal.pone.0201009)
Supplement: S2 Table — Aliquots of cell culture supernatant were collected and counted on the respective days. (DOCX) [file pone.0201009.s006.docx]

**S2 Table.** Cell counts for BBM.1 hybridoma during culture. Aliquots of cell culture supernatant were collected and counted on the respective days.

| **No. of days** | **Live Cells** | **Dead Cells** | **Live/Dead Cells** | **(10^6^) Live cells/mL** |
| --- | --- | --- | --- | --- |
| **2** | **78** | **7** | **11.1** | **0.39** |
| **5** | **124** | **89** | **1.39** | **0.62** |
| **9** | **46** | **72** | **0.64** | **0.23** |
| **12** | **33** | **113** | **0.29** | **0.17** |
| **16** | **50** | **174** | **0.29** | **0.25** |
| **19** | **22** | **212** | **0.10** | **0.11** |
| **23** | **10** | **161** | **0.06** | **0.05** |
| **26** | **0** | **173** | **0.00** | **0.00** |
