# Supplementary material for: Comparative validation of a microcapsule-based immunoassay for the detection of proteins and nucleic acids
Source: PLoS One. 2018 Jul 20;13(7):e0201009. doi: 10.1371/journal.pone.0201009 (PMC6054379; doi:10.1371/journal.pone.0201009)
Supplement: S3 Table — (DOCX) [file pone.0201009.s007.docx]

**S3 Table.** Normalized values of the hybridoma experiments performed with microcapsules and microplates with their respective concentrations, average and standard deviation.

1. **Detection of BBM.1 in PBS using microcapsules**

| **Conc (µg/mL)** | **N=1** | **N=2** | **N=3** | **AVG** | **STDEV** |
| --- | --- | --- | --- | --- | --- |
| 0.0000 | 0.34 | 0.26 | 0.44 | 0.35 | 0.09 |
| 0.0015 | -0.02 | 0.07 | -0.04 | 0.01 | 0.06 |
| 0.0046 | 1.16 | 0.66 | 1.14 | 0.99 | 0.28 |
| 0.0137 | -0.39 | 0.57 | 0.60 | 0.26 | 0.56 |
| 0.0412 | 0.05 | 0.04 | -0.09 | 0.00 | 0.08 |
| 0.1235 | 3.00 | 2.86 | 2.63 | 2.83 | 0.19 |
| 0.3704 | 20.03 | 19.02 | 18.89 | 19.31 | 0.62 |
| 1.1111 | 39.83 | 36.53 | 39.20 | 38.52 | 1.75 |
| 3.3333 | 70.24 | 63.02 | 65.39 | 66.21 | 3.68 |
| 10.0000 | 98.11 | 94.07 | 96.91 | 96.36 | 2.08 |
| 30.0000 | 102.30 | 95.81 | 101.88 | 100.00 | 3.63 |

1. **Detection of BBM.1 in PBS using microplate**

| **Conc (µg/mL)** | **N=1** | **N=2** | **N=3** | **AVG** | **STDEV** |
| --- | --- | --- | --- | --- | --- |
| 0 | 2.64 | -0.14 | -2.50 | 0.00 | 2.57 |
| 0.019 | 9.92 | 7.40 | 7.47 | 8.26 | 1.43 |
| 0.039 | 16.20 | 13.82 | 12.54 | 14.19 | 1.86 |
| 0.078 | 31.46 | 24.63 | 30.78 | 28.96 | 3.76 |
| 0.156 | 44.44 | 45.88 | 48.37 | 46.23 | 1.99 |
| 0.312 | 67.73 | 67.92 | 74.57 | 70.07 | 3.89 |
| 0.625 | 86.50 | 82.28 | 84.06 | 84.28 | 2.12 |
| 1.25 | 86.85 | 122.19 | 90.96 | 100.00 | 19.33 |
| 2.5 | 115.70 | 92.02 | 84.99 | 97.57 | 16.09 |
| 5 | 82.38 | 86.42 | 83.76 | 84.19 | 2.05 |
| 10 | 76.16 | 80.86 | 76.79 | 77.94 | 2.55 |

1. **Detection of BBM.1 in RPMI using microcapsule**

| **Conc (µg/mL)** | **N=1** | **N=2** | **N=3** | **AVG** | **STDEV** |
| --- | --- | --- | --- | --- | --- |
| 0.0000 | 0.23 | 0.15 | 0.03 | 0.14 | 0.10 |
| 0.0005 | 0.16 | 0.07 | 0.13 | 0.12 | 0.04 |
| 0.0015 | 0.05 | -0.01 | -0.04 | 0.00 | 0.05 |
| 0.0046 | 0.31 | 0.18 | 0.18 | 0.22 | 0.07 |
| 0.0137 | 1.46 | -0.90 | 1.37 | 0.65 | 1.34 |
| 0.0412 | 4.10 | 3.61 | 3.39 | 3.70 | 0.36 |
| 0.1235 | 20.48 | 18.14 | 17.90 | 18.84 | 1.43 |
| 0.3704 | 40.34 | 38.51 | 36.80 | 38.55 | 1.77 |
| 1.1111 | 63.86 | 63.86 | 62.21 | 63.31 | 0.95 |
| 3.3333 | 105.69 | 102.53 | 91.78 | 100.00 | 7.29 |
| 10.0000 | 97.23 | 97.62 | 100.52 | 98.46 | 1.80 |
| 30.0000 | 98.23 | 98.62 | 97.08 | 97.98 | 0.80 |

1. **Detection of BBM.1 in RPMI using microplat**

| **Conc (µg/mL)** | **N=1** | **N=2** | **N=3** | **AVG** | **STDEV** |
| --- | --- | --- | --- | --- | --- |
| 0.0000 | 1.05 | -0.99 | 1.85 | 0.64 | 1.46 |
| 0.0005 | 1.40 | 0.78 | -2.18 | 0.00 | 1.91 |
| 0.0015 | 0.75 | -0.62 | 0.77 | 0.30 | 0.80 |
| 0.0046 | -2.21 | 0.42 | 1.92 | 0.04 | 2.09 |
| 0.0137 | 3.16 | 0.88 | 4.10 | 2.71 | 1.66 |
| 0.0412 | 8.43 | 8.44 | 6.49 | 7.79 | 1.12 |
| 0.1235 | 17.08 | 20.67 | 24.71 | 20.82 | 3.82 |
| 0.3704 | 45.08 | 50.83 | 45.66 | 47.19 | 3.17 |
| 1.1111 | 78.33 | 74.89 | 78.81 | 77.34 | 2.14 |
| 3.3333 | 89.33 | 87.72 | 122.95 | 100.00 | 19.89 |
| 10.0000 | 87.12 | 87.50 | 87.20 | 87.27 | 0.20 |

1. **Detection of BBM.1 in hybridoma supernatant using microcapsule**

| **Conc (µL)** | **N=1** | **N=2** | **N=3** | **AVG** | **STDEV** |
| --- | --- | --- | --- | --- | --- |
| 0 | 0.09 | -0.05 | -0.04 | 0.00 | 0.08 |
| 0.007 | 16.47 | 16.02 | 14.45 | 15.64 | 1.06 |
| 0.03 | 26.63 | 26.87 | 28.80 | 27.43 | 1.19 |
| 0.12 | 35.27 | 36.14 | 32.50 | 34.64 | 1.90 |
| 0.49 | 42.09 | 42.42 | 39.35 | 41.28 | 1.68 |
| 1.95 | 54.17 | 51.31 | 47.81 | 51.10 | 3.19 |
| 7.81 | 94.91 | 89.09 | 92.00 | 92.00 | 2.91 |
| 31.25 | 85.48 | 85.97 | 83.95 | 85.13 | 1.06 |
| 125 | 86.63 | 83.27 | 88.98 | 86.30 | 2.87 |
| 500 | 95.21 | 95.81 | 88.18 | 93.07 | 4.24 |
| 2000 | 105.55 | 97.21 | 97.24 | 100.00 | 4.81 |

1. **Detection of BBM.1 in hybridoma supernatant using microplate**

| **Conc (µL)** | **N=1** | **N=2** | **N=3** | **AVG** | **STDEV** |
| --- | --- | --- | --- | --- | --- |
| 0 | 7.67 | 4.57 | 6.12 | 6.12 | 1.55 |
| 0.01 | 13.64 | 15.41 | 14.97 | 14.68 | 0.92 |
| 0.02 | 22.94 | 16.74 | 24.71 | 21.46 | 4.18 |
| 0.05 | 1.92 | 0.37 | 1.03 | 1.11 | 0.78 |
| 0.14 | 0.59 | -0.07 | -0.52 | 0.00 | 0.56 |
| 0.41 | 35.99 | 37.76 | 29.13 | 34.29 | 4.56 |
| 1.23 | 64.97 | 59.66 | 72.94 | 65.86 | 6.68 |
| 3.7 | 89.75 | 86.87 | 84.88 | 87.17 | 2.45 |
| 11.11 | 68.95 | 107.45 | 97.49 | 91.30 | 19.98 |
| 33.33 | 106.12 | 93.29 | 100.59 | 100.00 | 6.44 |
| 100 | 97.71 | 94.84 | 102.80 | 98.45 | 4.03 |
